# A Review on High-Power Ultrasound-Assisted Extraction of Olive Oils: Effect on Oil Yield, Quality, Chemical Composition and Consumer Perception

**DOI:** 10.3390/foods10112743

**Published:** 2021-11-09

**Authors:** Marco Nardella, Roberto Moscetti, Swathi Sirisha Nallan Chakravartula, Giacomo Bedini, Riccardo Massantini

**Affiliations:** Department for Innovation in Biological, Agro-Food and Forest Systems, University of Tuscia, 01100 Viterbo, Italy; marco.nardella@unitus.it (M.N.); rmoscetti@unitus.it (R.M.); swathi.nallan@unitus.it (S.S.N.C.); gbedini@unitus.it (G.B.)

**Keywords:** olive oil processing, malaxation, ultrasound, innovative technology

## Abstract

The objective of this review is to illustrate the state of the art in high-power ultrasound (HPU) application for olive oil extraction with the most recent studies about the effects of HPU treatment on oil yield, quality, chemical composition, as well as on the consumer’s perception. All the examined works reported an increase in oil yield and extractability index through the use of HPU, which was ascribed to reduced paste viscosity and cavitation-driven cell disruption. Olive oil legal quality was generally not affected; on the other hand, results regarding oil chemical composition were conflicting with some studies reporting an increase of phenols, tocopherols, and volatile compounds, while others underlined no significant effects to even slight reductions after HPU treatment. Regarding the acceptability of oils extracted through HPU processing, consumer perception is not negatively affected, as long as the marketer effectively delivers information about the positive effects of ultrasound on oil quality and sensory aspect. However, only a few consumers were willing to pay more, and hence the cost of the innovative extraction must be carefully evaluated. Since most of the studies confirm the substantial potential of HPU to reduce processing times, improve process sustainability and produce oils with desired nutritional and sensory quality, this review points out the need for industrial scale-up of such innovative technology.

## 1. Introduction

Mediterranean Diet (MD) as a concept and lifestyle emphasizes a specific dietary pattern that is typical of the Mediterranean basin, and particularly of the countries where the growth of olive trees is widespread, such as Greece, Crete, and Southern Italy [1]. The possible relationship between the adherence to MD and health benefits was proposed for the first time in 1634 CE by Lodovico Castelvetro, an Italian exiled in England who criticized the excessive consumption of meats by the English people, and wished for higher usage of fruits and vegetables [2]. It was not until the 1970s that the first large scale study was conducted on the relation of MD to health following a scientific approach, which showed lower rates of cardiovascular diseases (CVD) in Southern France, compared to Northern France [2] followed by many other studies on the benefits of MD [3,4,5]. The distinctive features of MD are that it is mainly a plant-based diet, characterized by a huge consumption of whole grains, fruits, and vegetables, moderate use of fish and white meat, and occasional consumption of red meat and sweets. In addition, a central trait is the use of olive oil as the main source of fat, which provides particularly monounsaturated fatty acids and a unique pool of micro-components, such as tocopherols and polyphenols [1].

Olive oil, its composition and, its positive effects on human health as a major component of MD have been thus extensively documented and reviewed in the literature [6,7,8,9,10]. A recent study conducted on mice by Luque-Sierra et al. [11] focused on the effects of supplementing their diet with extra-virgin olive oil (EVOO) containing a standard concentration of phenols. The results demonstrated that EVOO exerts vasculo-protective effects in mice aorta and reduces the expression of pro-inflammatory genes. Accordingly, mice fed with EVOO included diet exhibited atherosclerotic lesions of a lower extent, compared to those fed with the standard diet. Violi et al. [12] compared the effect of EVOO and corn oil on both glycemic and lipid profiles in healthy subjects. In this study, EVOO was observed to significantly reduce post-prandial glucose and low-density lipoprotein cholesterol levels, an effect that may be responsible for its anti-atherosclerosis action. Rubio-Senent et al. [13] ascribed this protective effect on CVD and, in particular, on the development of atherosclerotic plaques and the accumulation of oxidative damage, to the synergistic action of a consortium of phenolic compounds of EVOO. Moreover, EVOO phenols are involved in the modulation of the type-1 cannabinoid receptor gene (CB1) through epigenetic mechanisms which determine the suppression of colon cancer cells [14]. In fact, the leading factor in determining phenolic content and its composition in EVOOs is processing technology [15], which is strongly affected by traditions, probably limiting innovation aimed at improving product quality and the sustainability of the whole process.

Within the process flow of EVOO extraction, malaxation is crucial in determining both the yield and the overall quality of the product, especially in terms of micro-components, i.e., phenolic compounds and tocopherols concentration. This step consists of continually kneading the olive paste under controlled temperature and time conditions, which allows for a significant reduction of olive paste viscosity, facilitating the subsequent separation in the decanter [16,17,18]. Recent studies have been attempting to improve olive oil processing with particular attention towards the malaxation step, through the introduction of innovative technologies such as pulsed electric fields, microwaves, and ultrasound [19,20,21].

Among the various innovative technologies, ultrasound is particularly promising, with various applications in the food industry, such as improving process speed, the possibility to assist food drying, cutting, and emulsification, improving the extraction of bioactive compounds, inactivating food pathogens, and spoilage microorganisms, and to enhance or inhibit enzymatic activity depending both on processing parameters and type of enzymes [22,23,24,25,26,27,28,29,30,31,32,33], as briefed in Table 1.

The olive oil industry has also been adapting the application of ultrasound technology within the field of oil production, thanks to its effects on cell disruption. Among the various frequency ranges of ultrasound, high-power ultrasound (HPU) treatments in the frequency range of 18–40 kHz are observed to cause cell wall mechanical damages and disruption in vegetable tissues, thus promoting the release of soluble components and enhancing mass transfer [34,35]. The said effect can be explained by the cavitation phenomena taking place during the HPU treatment of food products. Briefly, upon HPU treatment cavitation, bubbles are produced in the vegetal tissues, which can increase in size until reaching a critical volume. This results in their collapse and leads to very high local temperature and pressure, which creates turbulence in the cavitation area named transient cavitation [36]. This physical effect plays a key role in the disruption of cells predominantly via cell wall damages, breaking, and pore formation in cell membranes [37]. Concerning EVOO extraction, cavitation can determine positive effects promoting the release of oil and micro-components, but could also have a negative impact due to excessive heating [16,17].

However, to the best of our knowledge, no attempts have been made to review recent literature on the effects of HPU on olive oil extraction and processing. Thus, the aim of this review is to summarize the main studies performed in the past five years (2016-till date) on the application of HPU in the extraction of olive oils and its effects on oil yield, legal quality, chemical composition, sensory attributes, and consumer perception.

The review consists of three sections: [i] the first section examines the effects of HPU on olive oil yield and extractability; [ii] the second one discusses HPU impact on olive oil quality and chemical composition, with a focus on oil legal quality parameters, phenolic content, lipid stability, volatile compounds, and sensory attributes, while [iii] the third section concerns consumer perception of olive oils extracted with the use of HPU.

## 2. Effect of HPU on Olive Oil Yield and Extractability

**Figure 1:**
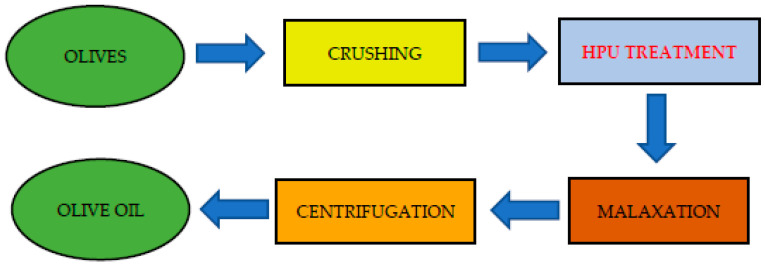
Flow chart of the olive oil production process with the HPU treatment implementation.

Many studies have investigated the potential application of HPU to olive paste immediately after olive crushing and before the malaxation step (Figure 1) with the objective of improving oil extraction yield from damaged cells [38,39,40,41,42,43,44]. Due to its mechanical and thermal effects attributed to acoustic cavitation, HPU provides the advantage of increasing both the efficiency and working capacity with the opportunity to remove the bottleneck represented by malaxation, which is an existing limiting factor for the continuity of the whole process [38].

Rigane et al. [43] underlined that a HPU treatment of olive paste at 35 kHz previous to malaxation led to improved oil yield (up to 20%, depending on experimental conditions), which reached the highest value after 10 min of treatment in the case of both the two studied olive varieties (i.e., Chemlali and Memecik). Whereas, Juliano et al. [42] explored the possibility of applying a double HPU treatment to olive paste immediately before and after malaxation, which allowed an additional recovery of oil with respect to the single treatment. In another study, Servili et al. [44] evaluated the importance of ultrasound pressure level in the treatment of olive paste between crushing and malaxation for four different olive cultivars. The results underlined the necessity to reach a pressure of 3.5 bar in order to increase oil extractability, while lower pressure treatments at 1.7 bar did not result in any significant improvement. The authors speculated that this difference could be ascribed to the higher mechanical impact of HPU with respect to low-pressure ultrasound. Actually, a previous study conducted on apples pointed out the possibility to identify an intensity threshold above which ultrasound-driven mass transfer is significantly improved according to the specific application and product [45]. A study by Iqdiam et al. [41] demonstrated that HPU treatment significantly increases (*p* ≤ 0.05) both oil yield and oil extractability index of 1.2 and 6.5%, respectively, compared to samples extracted through conventional malaxation. This improvement of oil extraction efficiency was probably related to HPU-driven lysis of cell membranes due to acoustic cavitation. In another study [40], the same author analyzed possible differences induced by HPU treatment time wherein, after 6 min of treatment the oil extractability index increased by 5.6–5.7%, while further sonication up to 8 or 10 min did not result in any improvement. The authors thus deduced that most cell damages are likely to occur within the first phases of treatment.

**Figure 2:**
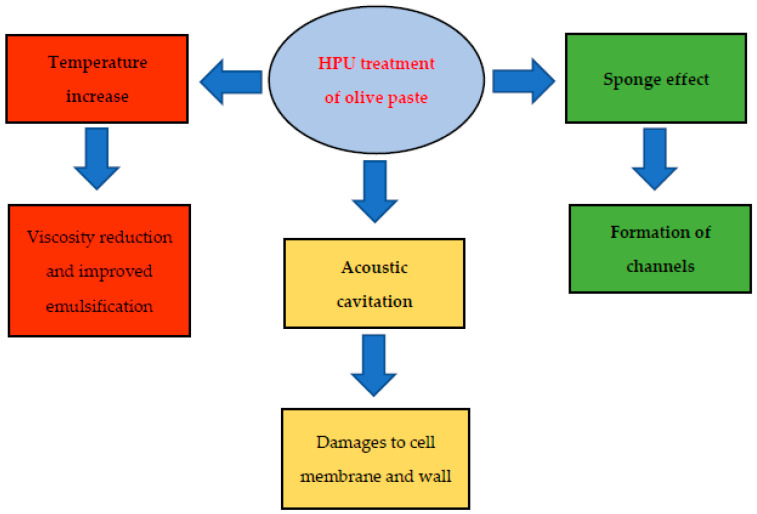
The main effects of olive paste HPU treatment contributing to improve oil yield.

Bejaoui et al. [16,39] obtained similar results, and the observed increase in oil yield was attributed to three main phenomena. Firstly, HPU determines a slight temperature increase of olive paste, resulting in decreased viscosity and better coalescence of oil droplets. This effect was particularly evident in the case of higher ultrasound frequencies. Secondly, the propagation of ultrasonic waves to a solid medium leads to a series of fast compressions and expansions of tissue which creates a movement described as the “sponge effect” [46]. This effect was observed to facilitate the release of liquids from damaged cells also via the creation of small channels. Thirdly, HPU-driven acoustic cavitation enhances the mass transfer in a solid-liquid extraction process through cavitation bubbles collapse, causing shear effects and shock waves, thereby provoking cell membranes microfractures and damages to the cell wall [39,47]. The different mechanisms through which HPU treatment improves olive oil yield, as discussed are summarized in Figure 2.

Moreover, HPU processing can be combined and applied simultaneously with other novel technologies like microwave, which significantly improves heat exchange and oil yield [48].

In conclusion, the examined literature indicates that the application of HPU to the olive oil production processing, and in particular, to assist malaxation, tends to significantly improve oil yield and increase the oil extractability index due to the coupled effects of both higher processing temperature and cavitation phenomena. However, considering the advantages and potential effects on chemical constituents, the HPU treatment should be tailored to achieve the best oil yield, without negatively affecting olive oil quality.

## 3. Effect of HPU on Olive Oil Quality and Chemical Composition

The objective of this section is to review the main literature on the impact of HPU treatment on olive oil parameters related with legal quality and chemical composition.

### 3.1. Legal Quality Indices

Olive oil legal quality is determined by a series of parameters, as defined by International Olive Council [49], which are used to determine olive oil quality category according to Regulation (EU) 2015/1830 [50]. Among these parameters free acidity, peroxide value, and spectrophotometric indices (K_232_ and K_270_ concerning primary and secondary lipid oxidation, respectively) are the most relevant in the literature concerning HPU processing of olive paste. None of these parameters seem to be affected by the treatment [39,40,41,42,44,51,52]. Just one study reported a slight but significant increase of peroxide value as a result of HPU treatment. However, such increase was underlined only in the case of treatments longer than 6 min and did not represent an issue for the labeling of olive oil as EVOO [40].

### 3.2. Total Phenolic Content

Phenolic compounds present in olive oil are responsible for potent effects on health, as well as improvement of sensory aspect by enhancing bitter and pungent notes [6,53,54] and are usually affected by processing and storage conditions.

In this regard, the results on comparison between phenolic content of oils produced through conventional and HPU-assisted extraction published in literature are conflicting. In fact, most studies reported an increase in the concentration of phenols achieved through HPU treatment [51,55,56]; whereas several others did not underline significant effects on phenols extraction [57,58], while another study stressed a reduction in the concentration of these compounds [40].

The increase of total phenolic compounds observed by Almeida et al. [59], who applied HPU after malaxation, was justified by the collapse of acoustic cavitation bubbles, leading both to cell wall disruption, and therefore better release of phenolics from the fruit mesocarp, and improvement of emulsifying capacity. Taticchi et al. [51] observed an increase of approximately 10% of the phenolic content in oils extracted with HPU, which was attributed to the enhanced cell degradation.

Another main factor that could account for the improvement of phenolic content through olive paste HPU treatment is the inactivation of poly-phenoloxidase (PPO), which is responsible for the oxidative losses of phenolic compounds during malaxation [55]. However, PPO activity could also be increased during relatively short HPU treatments of lower power since PPO works better as soon as the paste starts to be heated [58].

Iqdiam et al. [40] noted a reduction of phenolic content after HPU treatment (approx. 20%) ascribed to ultrasound-induced heating of the olive paste and to shear forces possibly provoking changes in the phenols chemical structure.

**Figure 3:**
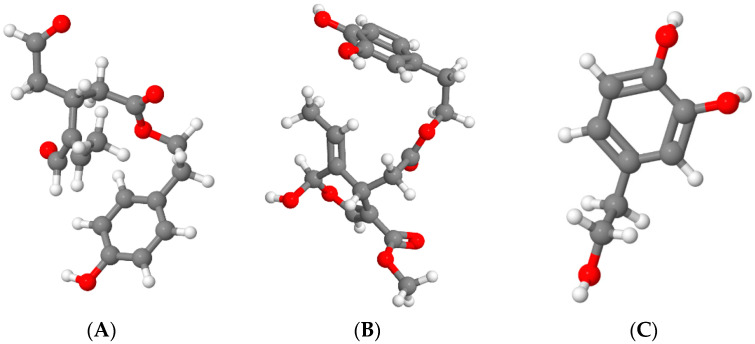
Chemical structures of (**A**) p-HPEA-EDA, (**B**) 3,4-DHPEA-EA, and (**C**) hydroxytyrosol. The images were obtained with Jmol viewer [60].

However, where observed, the increase in phenols concentration did not involve all phenolic compounds, but mainly some secoiridoid derivatives such as p-HPEA-EDA and 3,4-DHPEA-EA, which are strictly related to hydroxytyrosol [55,57,58]. This could be explained by the fact that the final concentration of these molecules is the result of a complex mixture of mechanical, biochemical (enzymatic activity), and chemical (equilibrium between aqueous and oily phase) reactions which are strictly affected by processing parameters (power, time and temperature of the treatment) [56]. The chemical structures of p-HPEA-EDA, 3,4-DHPEA-EA, and hydroxytyrosol are represented in Figure 3.

The effect of temperature is particularly debatable: on one hand, higher temperatures might determine the degradation of phenolic compounds and enhance PPO activity leading to phenols degradation whereas, on the other hand, high temperatures could improve the diffusion and solubility of polyphenols resulting in enhanced extraction rates [58]. Ultrasound power and treatment time are the main parameters involved in olive paste heating. An experiment performed through response surface methodology confirmed the rigorous relationship existing between phenolic concentration and the temperature reached during HPU treatment [61].

Furthermore, there are other factors not connected with HPU application that might determine important differences in the phenols content of the extracted oils. The most important factor above all is the oxygen concentration in the headspace during malaxation since it plays a key role in many enzymatic and non-enzymatic oxidation reactions [52]. The variability in the fruit used depending on olive cultivar, geographical origin, seasonality, and harvesting time is another main factor that should be considered, as not all phenolic compounds undergo the same changes during HPU treatments [57].

Finally, the different analytical techniques used for total phenols determination should be considered since they could affect the final result. In fact, in some of the cited studies, total phenolic content was determined through the Folin-Ciocalteu method, while in others as a sum of the areas of the chromatographic peaks found through HPLC analysis. This is clearly a significant factor because it is possible that not all phenolics were included. Moreover, some interferents were highlighted in the case of Folin-Ciocalteu assay, such as ascorbic acid [62], possibly leading to an overestimation of total phenol content. These aspects make more difficult a comparison between the different studies.

### 3.3. Lipid Stability and Fatty Acid Composition

Lipid oxidation is the principal factor limiting the shelf life of olive oils, which are generally quite stable to autoxidation phenomena due to the high content of monounsaturated fatty acids and minor compounds, i.e., phenolics and tocopherols showing antioxidant activity [16,63]. HPU treatment of olive oils does not seem to significantly modify their oxidative stability [16,39,63]. However, few works underlined some differences between the lipid oxidation rates of oils obtained through conventional and HPU-assisted extraction. Specifically, Rigane et al. [43] highlighted an HPU-driven increase in the induction time which was justified through the enhanced content of antioxidants that were more easily extracted from the paste after HPU treatment. In contrast, Iqdiam et al. [40] registered a decrease in the induction time attributed to paste heating during HPU treatment (20 kHz), leading to the degradation of micro-components with antioxidant activity and to lipid autoxidation [64]. However, this decrease in lipid stability was observed only in the case of treatments longer than 8 min, while no significant effects were observed for shorter treatment times. This corroborates the importance of processing parameters on paste heating, including ultrasound power and treatment time, thereby justifying the observed discrepancies in literature.

HPU treatment of high-unsaturated fatty acid foods is generally problematic since those fatty acids are more susceptible to autoxidation [65]. Nevertheless, Chemat et al. [66] applied HPU to the extraction of various vegetable oils at a temperature lower than 20 °C demonstrating that this drawback mainly involves oils with a high content of polyunsaturated fatty acids (e.g., sunflower and corn oils). In fact, they were severely degraded due to HPU, developing off-flavors. On the contrary, oils rich in monounsaturated fatty acids (olive and peanut) were not affected.

With regard to olive oil fatty acid composition, it is generally not affected by HPU treatment [57,63]. Bejaoui et al. [57] observed some significant differences between HPU and conventionally extracted oils, but they concluded that such discrepancies were likely due to the variability in fruits of Picual variety, since lipid composition is affected also by agronomical practices [67].

### 3.4. Volatile Compounds and Sensory Properties

Most of the volatile compounds that characterize olive oils are produced during malaxation due to the activation of specific pathways, among which the lipoxygenase enzyme (LOX) takes on great importance generating a wide range of C6 aldehydes, alcohols, and esters, accounting for most of the positive sensory notes of olive oils. Such modifications are triggered when olive fruit tissues are damaged, thus facilitating the release of endogenous enzymes such as lipoxygenase and hydroperoxide lyase [68].

In this regard, Bejaoui et al. [39,57] studied the effect of HPU-assisted extraction on the concentration of olive oil volatile compounds and concluded that HPU treatment did not alter its volatile profile in terms of both LOX and non-LOX products. The pungent, bitter, and fruity sensory notes of the oils were not negatively affected by the treatment, and the produced oils showed a more harmonic and equilibrated profile.

Other studies pointed out some differences between the volatile and sensory profiles of oils obtained through conventional and HPU-assisted extraction. Almeida et al. [59] observed an improved activity of the enzymes belonging to the LOX pathway after HPU treatment, leading to an increase of C5 alcohols. This result was justified by HPU-driven modulation of endogenous enzymes, through changes in protein conformation and, as a result, an increased affinity for substrates. Thus, the treatment determined a marked increase in pungency and bitterness of the oils, because of the improved phenolic content and fruitiness, due to the increased activity of LOX enzymes.

A decrease in the concentration of volatile compounds after HPU treatment could also occur, depending on processing conditions, i.e., ultrasound power and treatment time. A decrease in C5 and C6 aldehydes of approx. 5% following HPU treatment was reported by Taticchi et al. [51], which was ascribed to partial inactivation of the enzymes belonging to the LOX pathway as a result of the physical effects of cavitation. In this regard, a key parameter is the treatment time, for which a recent study [59] underlined no significant changes in the volatile fraction up to 30 min of HPU treatment at 40 kHz, while a significant decrease was observed after 60 min due to further heating of olive paste during malaxation.

### 3.5. Other Physico-Chemical and Chemical Characteristics

Regarding total tocopherols, some studies report an increase achieved with HPU treatment (approx. 20%), mainly involving α-tocopherols (up to 50%) [40,55]. This effect was caused by acoustic cavitation, which improves mass transfer in both liquid and solid foods. In fact, in HPU-treated solid media, both internal and external mass transfer is improved by the dissipation of acoustic energy and ultrasonic waves-induced mechanical stirring, respectively. The former leads to temperature rise, while the latter reduces boundary layer thickness [69]. However, also other mechanisms seem to be involved, such as the generation of microjets due to the asymmetric implosion of acoustic cavitation bubbles [45] and the formation of microchannels decreasing the resistance to water transfer [26]. On the other hand, a work by Almeida et al. [59] highlighted a decrease in total tocopherols after HPU treatment. The authors concluded that these compounds could have undergone ultrasound-induced degradation by thermolysis, or oxidation phenomena triggered by the generation of free radicals during the treatment.

The concentration of pigments (i.e., carotenoids and chlorophylls) is positively affected by HPU application [40,51,55] with an increase in percentage, which mainly depends on the extent of cell membrane damages achieved through the treatment. It is likely that this widely documented increase in pigments, together with the reduction of oil turbidity level achieved with HPU treatment of the paste, determines the color changes observed by Taticchi et al. [51]. They noted that oils extracted through HPU had higher values of lightness (L*) and higher intensity of greenness (a*) and yellowness (b*). The Euclidean distance (ΔE*) between oils produced through conventional and HPU-assisted extraction in the CIELab color space, was 5.76 indicating that the color difference can be easily perceived by the human eye.

In conclusion, the impact of HPU on olive oil quality and chemical composition is not univocal, especially in the case of micro-components, i.e., polyphenols, tocopherols, and volatile compounds. However, these contrasting results primarily depend on the complexity of physical and biochemical mechanisms involved in the transformations occurring during olive oil production and on the wide variety of processing parameters, such as ultrasound power, temperature, and time of treatment, as well as on variables that are not strictly related with HPU application, such as oxygen concentration in the headspace during malaxation, olive variety and climate. Thus, a HPU treatment could be designed in order to improve the final content of antioxidants and volatile compounds through enhanced extraction, limiting oxidative and thermal losses. As many discrepancies were found in the literature regarding the effects of HPU on olive oil nutritional and sensory quality, Table 2 is an attempt to resume the results observed in the principal studies.

## 4. Consumer Acceptance of Oils Extracted through HPU

The traditional character of EVOO is probably the main factor limiting the introduction of innovative technologies in this field, such as HPU processing. The objective of this section is to determine whether consumers could be open to such innovation and willing to pay more to obtain a more sustainable product that naturally contains a higher concentration of micro-components which could benefit human health and improve oil shelf-life.

As highlighted by Guerrero et al. [70], when innovations are applied to traditional food products, the degree of acceptance strongly depends on the perceived benefits which could be achieved, i.e., improvement of nutritional and sensory quality. In this context, Cavallo et al. [71] underlined that the claim “extracted through ultrasound” did not negatively affect consumers’ acceptance of EVOO but they were not able to conclude that consumers could be willing to pay more to get a higher quality product.

Another study carried out by Roselli et al. [72] attempted to highlight the principal factors affecting the willingness of consumers to buy EVOOs extracted with ultrasound through a questionnaire. The results of the study demonstrate that the main element involved is the consumer perception generated by the fact that such technology could eventually improve product quality, followed by the enhancement of sensory attributes mainly through the improvement of fruity notes. Regarding the population characteristics, education level seems to play the greatest role: in general, the higher the education level, the higher the consumer awareness about the advantages and drawbacks of an innovative process was. However, about half of the consumers were willing to purchase HPU-extracted EVOO, while only 9% were willing to pay an additional price to buy it. In a recent study [73], in order to achieve higher acceptance rates for the product, the same author performed a Latent Class Model analysis to define possible market segments to which HPU-extracted EVOO could be addressed. The results revealed three segments defined as follows: innovative (32%), those that are most open to innovations in EVOO processing and like sweet and fruity notes; traditionalist (25%), those being the most hesitant to such technologies; cautious (43%), having no statistically significant repulsion for EVOOs extracted through HPU. The first cluster (innovative) was proven to appreciate and prefer HPU-extracted EVOOs compared to the ones obtained with conventional extraction. The respondents belonging to such group tended, on average, to pay more attention to executing healthy and ethical food choices and could therefore represent the target to which innovative EVOOs should be addressed.

In the light of the literature research, it seems clear that the most important aspect to achieve a higher acceptance rate for HPU-extracted olive oils is to create a positive perception of the product, highlighting the positive effects of such technology, in terms of waste reduction and improvement in antioxidants content. HPU-extracted olive oils should be addressed to a specific market segment (niche market), with consumers of higher awareness levels and open to innovation, since it is much more likely that they are also willing to pay more to get a higher quality product.

## 5. Conclusions

The reviewed literature confirms that HPU treatment could be successfully implemented in EVOO processing to enhance, shorten or substitute malaxation step, or eliminate olive paste pre-heating. There is evidence that HPU-driven extraction of olive oil leads to higher yields and improves oil extractability index through reduced paste viscosity and acoustic cavitation phenomena provoking cell membrane damages. Such treatment might also affect the development of phenolic and volatile compounds and consequently olive oil health properties, oxidative stability, and sensory aspect (pungent, bitter, and fruity notes). Nevertheless, the treatment should be carefully designed to produce olive oils with the desired characteristics since too high ultrasound power and/or too long treatment times might detrimentally affect the stability of EVOO components. Further research is needed to better highlight the main processing parameters to modulate for obtaining oils with tailored physico-chemical, chemical, and sensorial properties. As for the consumer perception, olive oils extracted through HPU are generally accepted, but only a small proportion of consumers are willing to pay an additional price for such a high-quality product. This percentage largely depends on the ability to deliver information regarding the advantages of the innovative process, i.e., health benefitting properties (phenols enrichment) and sustainability (waste reduction), which could help to overcome the skepticism generated by the non-traditional process. In view of this, the costs of the innovative extraction process should also be analyzed and compared to the traditional one to determine whether a significant competitive advantage provided by the HPU application can be highlighted.

## Figures and Tables

**Table 1:** The main applications of ultrasound in the food industry.

Application	Food Matrix	Ultrasound Power	References
Cooking velocity	Mortadella	301 W	[22]
Freezing speed up	Potatoes	270 W	[23]
Fruit drying/dehydration	Bananas/Apples	4000/4870 W/m^2^	[26,27]
Cutting	Cheese	750 W	[28]
Filtration	Grape pomace extract	400 W	[29]
Emulsification	Nanoemulsions	450 W	[30]
Bioactives extraction	Pomegranate/Tea	400/600 W	[31,32]
Microbial inactivation	Apple juice	600 W	[33]
Enzyme inactivation/modulation	Carrots/Lipases	400 W/2–21 W/cm^2^	[24,25]

**Table 2:** Impact of HPU-assisted extraction on olive oil phenolic and volatile compounds concentration, as compared to traditional extraction procedure.

Olive Cultivar	Ultrasound Frequency (kHz)	Treatment Time (min)	Phenolic Compounds	Volatile Compounds	References
Picual	40	10–40	↓	↔	[39]
Ogliarola	20	10–30	↑	↓	[51]
Picual	20–80	30	↑	↔	[57]
Carolea	25	15	↑	↑	[59]
Arbequina	40	10–60	↓	↓	[63]
Picual			↔	↓	

↑ Increased concentration; ↓ decreased concentration; ↔ no significant differences.

## Data Availability

No new data were created or analyzed in this study. Data sharing is not applicable to this article.
